# Impact of hypertension on various markers of subclinical atherosclerosis in early type 2 diabetes

**DOI:** 10.1186/2251-6581-13-24

**Published:** 2014-01-29

**Authors:** Sudabeh Alatab, Hossein Fakhrzadeh, Farshad Sharifi, Ali Mostashfi, Mojde Mirarefin, Zohreh Badamchizadeh, Yaser Tagalizadehkhoob

**Affiliations:** 1 Elderly Health Research Center, Endocrinology and Metabolism population Sciences Institute, Tehran University of Medical Sciences, North Karegar Avenue, Dr Shariati Hospital, 5th floor, Tehran 1411413137, Iran; 2 Radiology Departement, Khatamolanbia hospital, Tehran Iran

**Keywords:** Diabetes melitus, Subclinical atherosclerosis, CIMT, FMD, ABI, Hypertension

## Abstract

**Background:**

Presence of Diabetes Mellitus increases the risk of subclinical atherosclerosis. In this study was aimed to determine the influence of hypertension (HTN) on surrogate markers of atherosclerosis in a population of patients with early type 2 diabetes.

**Methods:**

125 diabetic subjects drawn from Dr. Shariati outpatient’s clinic list and 153 non- diabetic subjects who were the relatives in law of diabetic participants were recruited. Participants with type 2 diabetes were free of clinical evidence of cardiovascular disease and renal involvement. Two groups of diabetic and control were further divided into two subgroups of hypertensive (known case of HTN or blood pressure ≥140/90 mmHg) and normotensive, and anthropometric characteristics, metabolic biomarkers as well as markers of subclinical atherosclerosis including Carotid intima media thickness (CIMT), flow mediated dilation (FMD) and Ankle Brachial Index (ABI) were measured.

**Results:**

Diabetic group with a mean age of 49.9 ± 7.5 years had significantly higher CIMT (0.64 ± 0.14 vs 0.76 ± 0.19, p = 0.001) and lower FMD (16.5 ± 8.1 vs 13.3 ± 7.1, p = 0.003) and ABI (1.2 ± 0.1 vs 1.1 ± 0.1, p = 0.01) than control with mean age of 52.9 ± 10.1 years. 34% of control and 59.2% of diabetic were hypertensive. Fasting blood sugar, insulin levels and calculated insulin resistance index of HOMA IR. of hypertensive subjects were higher than normotensive subjects in both groups of diabetic and non-diabetic. Similar pattern was presented for measured inflammatory mediators of hs-CRP and IL-6. Among subclinical atherosclerosis markers, only CIMT was significantly different between hypertensive and normotensive subjects in both groups. In adjusted linear regression analysis, a constant significant association existed between age and CIMT, ABI and FMD in non-diabetic, while in diabetic, age only correlated with CIMT and not the other two markers. In multiple regression model, HTN was recognized as a risk factor for increasing CIMT (OR = 2.93, 95% CI = 1.03-8.33, p = 0.04) but not attenuating FMD or ABI.

**Conclusions:**

Since FMD and CIMT may measure a different stage of subclinical atherosclerosis in diabetic patients, influence of HTN on these markers might be different.

## Introduction

Type 2 diabetes mellitus (T2DM) is a chronic disease that comprises an array of dysfunctions resulting from the combination of resistance to insulin action and inadequate insulin secretion. Patients with diabetes are in need of continuous medical care and self-management education to prevent acute complications and reduce the risk of long-term complications. Worldwide, the number of patients with diabetes is increasing mostly because of aging, urbanization, and increased prevalence of obesity and physical inactivity. Beside the severe microvascular complications, patients with T2DM, are at increased risk of macrovascular complications including coronary artery disease (CAD) [1,2]. The process of accelerated and premature atherosclerosis in diabetic leads to an increased risk of cardiovascular events. It has been shown that diabetic subjects are twice as likely to have a heart attack or stroke. Indeed, myocardial ischemia, caused by atherosclerotic involvement of coronary arteries, more frequently occurs without prior symptoms in these patients. In fact atherosclerotic involvement of vessels is often present before ischemic symptoms occur and before treatment is started. Early recognition of asymptomatic atherosclerosis in diabetes would be certainly important in reducing the risk of diabetic macrovascular complication and improvement of the prognosis. Clinical manifestations of atherosclerosis occur mainly in coronary arteries, lower extremity arteries, and carotid arteries.

Atherosclerosis can be assessed using non-invasive techniques, such as carotid intima-media thickness measurement (CIMT) [3], brachial artery flow-mediated dilatation test (FMD) [4] and ankle-brachial index (ABI) [5,6].

CIMT as measured by B-mode ultrasound represents the combined thickness of the intimal and medial layers of the carotid artery and represents an important predictive factor which favorably correlates with the risk of myocardial infarction and stroke, even after excluding the impact of other cardiovascular disease risk factors [7].

Endothelial dysfunction, characterized by a reduced bioavailability of endothelium-derived nitric oxide, is probably one of the earliest components of atherosclerosis [8]. Presence of endothelial damage as defined by abnormal FMD levels predicts adverse cardiovascular outcomes [9].

The ABI, which is the ratio of systolic pressure at the ankle to that in the arm, is a simple, inexpensive and useful method for assessing peripheral artery disease. Meta-analyses of large observational studies with long-term follow-up have reported that ABI is associated with coronary heart events independent of traditional Framingham variables [10].

Several factors have been proved to affect the risk of atherosclerosis, among them hypertension is a well-established- traditional risk factor. Clinical trials have shown that, in the highest quintile of diastolic pressure, even with the added risks of high cholesterol and smoking, hypertension still contributes significantly to risk for atherosclerosis [11]. Considering the fact that atherosclerosis is also an accelerated process in diabetic subjects we aimed to assess the hypertension – associated additive vascular effects on surrogate markers of atherosclerosis in diabetic subjects in the absence of other cardiovascular risk factors. In this regard the impact of presence or absence of hypertension on endothelial function (measured by FMD), and structure (measured by CIMT), and also on peripheral arterial disease (measured by ABI) was assessed in subjects with and without diabetes.

## Methods and materials

### Subjects

This case- control study was performed in the Endocrinology and Metabolism Research Institute (EMRI) of Tehran University of Medical Sciences. A total of 125 diabetics and 153 non- diabetic subjects as control group were recruited in this study. The participants were drawn from the list of diabetic patients who attended diabetes outpatient clinic at Dr. Shariati Hospital; Tehran, Iran; for regular follow-up between January to August 2010. The control group was composed of healthy subjects.

The inclusion criteria were defined as both sexes, aged between 20-65 years. Current or previous smokers, subjects with symptomatic or previous history of cardiovascular disease, myocardial infarction, diabetic foot, renal failure (GFR < 90 cc/min), known case of malignancy, cirrhosis and other severe disorders were excluded from the study. All the participants had undergone the Exercise stress test based on Bruce exercise protocol with negative result.

For all diabetic participants retinoscopy was performed by two ophthalmologists. The patients with proliferative retinopathy were excluded from the study. Peripheral neuropathy was diagnosed using the 10-gram Semmes–Weinstein monofilament test on the plantar surface of the feet in diabetic patients. Patients who could not reliably sense the filament were considered as diabetic neuropathy and excluded from the study.

In all the participants, fasting plasma sugar (FBS) and 2-h post load glucose were measured. Diabetes mellitus was defined as patients who were using either oral hypoglycemic agents or patients who had a FBS ≥ 126 mg/dl, or a 2-h post load glucose ≥ 200 mg/dl [12]. Hypertensive patients were those who were using anti-hypertensive agents or having a systolic blood pressure of ≥ 140 or diastolic blood pressure ≥90 mmHg according to The Seventh Report of the Joint National Committee on Prevention, Detection, Evaluation, and Treatment of High Blood Pressure guideline [13].

All of the participants underwent physical examination and anthropometric evaluations at the time of recruitment. Height was measured with a Stadiometer Height measurement device. Weight was measured on a calibrated beam balance. Blood pressure was measured twice (5 minutes interval) using a standard calibrated mercury sphygmomanometer on both right and left hands after the participants had been sitting for at least 10 minutes. The highest blood pressure of two sides was considered as the participant’s blood pressure. The protocol of study was approved by the ethics committee of Tehran University of Medical Sciences and the participants signed their informed consent at the time of recruitment.

### Laboratory measurement

Venous blood samples were collected in the morning after 8-12 h of fasting. The blood samples were centrifuged and then serum was collected.

Plasma levels of glucose, triglyceride (TG), total cholesterol, high density lipoprotein (HDL) cholesterol, low density lipoprotein (LDL) cholesterol, LDL cholesterol, creatinine, blood urea nitrogen (BUN) and high sensitive C-reactive Protein (hs-CRP) were measured by a colorimetric method using Pars Azmoon kit with an auto-analyzer (Autoanalyzer Hitachi 902). Serum Insulin concentration was assessed by immunoassay (ELISA) using a Bioscience kit (Monobind kit USA). Plasma Homocysteine and HbA1c were detected by High-performance Liquid Chromatography (HPLC) (KNAUER, Germany), coupled with fluorescence detector. The Serum IL-6 was measured by ELISA (R&D system, USA). The intra and inter-assay coefficients of variation (CVs) for all these measurements were less than 4% which was less than allowed CVs. A morning clean catch midstream urine sample was collected for evaluation of microalbuminurria.

Homeostasis model assessment (HOMA) index was calculated as the product of the fasting plasma insulin level (μ IU/mL) and the fasting plasma glucose level (mmol/L), divided by 22.5.

### Cardiovascular and neurologic evaluations

#### CIMT

Ultrasonographic analysis of the carotid artery was performed with a high-resolution ultrasound scanner, equipped with a linear array 13 MHz transducer (MyLab 70 XVision, biosound esaote USA) as we reported in our previous study [14]. Briefly, a rapid cross sectional scanning followed by a longitudinal scanning of the common carotid artery (CCA) was made in the first step to pinpoint the possible plaques. Then the dynamic sequence images were stored for measurement of CIMT. The CIMT was defined as the distance between the leading edge of the lumen-intima interface and the leading edge of the media-adventitia interface. For precise measurement of CIMT software (Vascular Tools 5, Medical Imaging Applications LLC, USA) was employed. The regions of interest were defined as 1.0 cm distal to the bifurcation, the bifurcation and 1.0 cm proximal to the internal carotid artery in both near and far walls. The CIMT was reported for each subject as the average of 12 measurements (6 measurements from the right and 6 from the left carotid artery).

### FMD

FMD of the brachial artery was measured according to the American College of Cardiology guidelines [15] and as we reported previously [14]. The diameter of the right brachial artery was measured 3–5 cm above the antecubital fossa. Then a blood pressure cuff was inflated around the right forearm to at least 50 mmHg above the systemic blood pressure for 4-5 minutes. 60 seconds after cuff release, the diameter of the brachial artery was measured. The brachial FMD was calculated as the percentage of change in the maximum post-occlusion diameter of the brachial artery relative to the mean baseline diameter. All the measurements were performed in the end-diastolic phase coinciding with the R-wave on an electrocardiograph monitor. Every measurement was taken as the average of 3 consecutive cardiac cycles.

### ABI measurements

For calculating the ABI, the systolic blood pressure from both brachial, dorsalis pedis and posterior tibial artery, was assessed while the subject stayed in the supine position for 10 minutes using a handheld Doppler. The highest blood pressure recorded in left or right sides of dorsalis pedis or posterior tibialis arteries was considered as numerator of ABI and the highest blood pressure of right or left side brachial artery was considered as denominator of ABI.

### Data analysis

For data analysis the SPSS (version 18.0) was used. Normality of data distribution was evaluated by Kolmogrov-Smirnov test. For data without normal distribution, Mann-Whitney U test was applied. For comparing data with normal distribution independent-samples T test was used. Adjustment for confoundin factors was performed using univariate analysis of variance (ANOVA). Correlation of variables was demonstrated using Pearson’s and Spearman’s correlation coefficients in normally distributed parametric and nonparametric variables respectively. For assessment of association of variables, linear regression and logistic regression were used for parametric and binary variables respectively.

## Results

278 subjects (125 diabetic and 153 control subjects) were enrolled in this study. Two groups had significant difference in respect to age, lipid and glucose profile (Table 1). Diabetic subjects presented with significantly higher levels of hs-CRP compared to control group. 34% (n = 52) of control group and 59.2% (n = 74) of diabetic subjects were hypertensive (p = 0.001). The means of three measured subclinical atherosclerosis indexes of CIMT, FMD and ABI were remarkably different between two groups of diabetic and control. These difference stayed significant even after age and sex adjustment in multivariate analysis (P = 0.013 for ABI, P = 0.0001 for CIMT and P = 0.01 for FMD).

**Table 1:** Anthropometric and clinical characteristics of control and diabetic subjects

**Variables**	**Control (n = 153)**	**Diabetic (n = 125)**	**p**
Age (year)	49.9 ± 7.5	52.9 ± 10.1	0.001
Female (%)	58.3	53.6	0.4
Hypertensive subjects (%)	34	59.2	0.01
Hypertension duration (year)	0.9 ± 0.3	2.0 ± 0.7	0.001
Waist circumference (cm)	92.2 ± 10.8	94.7 ± 10.7	0.06
BMI (kg/m2)	28.5 ± 4.4	27.9 ± 4.3	0.2
Systolic BP (mmHg)	125.7 ± 16.7	134 ± 17.6	0.001
Diastolic BP (mmHG)	77.9 ± 10.7	78.2 ± 10.2	0.8
Duration of DM (year)	0	8.7 ± 6.6	0.001
Cholesterol (mg/dl)	199.9 ± 34.2	176.7 ± 41.0	0.001
Triglyceride (mg/dl)	161.4 ± 78.6	192.8 ± 107.4	0.006
HDL cholesterol (mg/dl)	46.5 ± 10.6	42 ± 10	0.001
LDL cholesterol (mg/dl)	113.6 ± 23	95.7 ± 25.4	0.001
FBS (mg/dl)	94.7 ± 12	164.1 ± 61.2	0.001
Insulin (IU/L)	8.6 ± 3.4	8.5 ± 4.5	0.9
HbA1C	5.3 ± 0.6	7.9 ± 1.7	0.001
HOMA IR	2.1 ± 1.4	3.5 ± 2.8	0.001
Creatinine	0.9 ± 0.1	0.9 ± 0.2	0.8
Hs-CRP	2.3 ± 2.6	3.3 ± 2.9	0.03
IL-6	4.5 ± 5	5.4 ± 4.8	0.1
CIMT	0.64 ± 0.14	0.76 ± 0.19	0.001
FMD%	16.5 ± 8.1	13.3 ± 7.1	0.003
FMD <5.5 n, %	4, 3.5%	14, 13.9%	0.01
FMD ≥5.5 n, %	110, 96.5%	87, 86.1%	0.01
ABI	1.2 ± 0.1	1.1 ± 0.1	0.01

The data are presented as mean ± SD; comparison of variables means in two groups performed by Student T test; BMI = body mass index; BP = blood pressure; HDL = high-density lipoprotein; LDL = low-density lipoprotein; FBS = fasting blood sugar; HOMA IR = Homeostatic model assessment; hs-CRP = high sensitive C-reactive protein; IL-6 = Interleukin 6; CIMT = Carotid Intima Media thickness; FMD = Flow Mediated Dilatation; ABI = Ankle - Brachial Index.

We subdivided the two groups of diabetic and control groups into two subgroups of hypertensive and non-hypertensive (Table 2). In control group, the hypertensive subjects were significantly older and had greater BMI. Interestingly, FBS, insulin level and HOMA.IR were higher in this subgroup. Similarly the diabetic hypertensive subjects were older and had greater levels of both LDL-cholesterol and cholesterol.

**Table 2:** Characteristics of normotensive and hypertensive subjects in control and diabetic groups

**Variables**	**Non-hypertensive (n = 101)**	**Hypertensive (n = 52)**	**p**	**Non-hypertensive (n = 51)**	**Hypertensive (n = 74)**	**p**
Age (year)	50.6 ± 12.56	54.5 ± 7.8	0.04	48.7 ± 7.39	52.2 ± 7.1	0.00
Female (%)	47.1	58.1	0.27	62.4	51.9	0.22
Waist circumference (cm)	92.86 ± 7.31	96.03 ± 12.37	0.07	90.71 ± 10.89	95.21 ± 10.15	0.01
BMI (kg/m2)	26.48 ± 3.74	28.93 ± 4.33	0.00	28.49 ± 4.75	28.64 ± 3.85	0.85
Systolic BP	122.04 ± 9.90	142.26 ± 17.07	0.00	117.91 ± 11.08	140.63 ± 1563	0.00
Diastolic BP	72.25 ± 7.92	82.36 ± 9.72	0.00	73.56 ± 7.62	86.25 ± 10.77	0.00
Duration diabetes (year)	0	0		10.07 ± 7.10	6.73 ± 5.40	0.00
Cholesterol (mg/dl)	171.00 ± 37.37	180.59 ± 43.16	0.20	194.99 ± 32.16	209.43 ± 36.31	0.01
Triglyceride (mg/dl)	158.4 ± 87.05	167.31 ± 59.20	0.01	168.2 ± 88.49	209.26 ± 11.12	0.07
HDL cholesterol (mg/dl)	41.33 ± 10.25	42.41 ± 9.78	0.55	45.94 ± 10.06	47.53 ± 11.52	0.38
LDL cholesterol (mg/dl)	92.65 ± 22.72	97.74 ± 27.06	0.27	110.33 ± 21.73	119.90 ± 24.17	0.01
FBS (mg/dl)	93.10 ± 11.41	97.80 ± 12.64	0.02	155.10 ± 49.89	170.19 ± 67.45	0.17
Insulin (IU/L)	6.20	7.25	0.05	6.75	8.30	0.04
HOMA IR	1.93 ± 1.39	2.34 ± 1.25	0.04	2.84 ± 1.25	3.89 ± 2.9	0.09
Hs-CRP	2.11 ± 2.05	2.59 ± 3.37	0.28	2.88 ± 4.6	3.64 ± 5.1	0.4
IL-6	3.28 ± 1.82	3.93 ± 2.98	0.12	4.44 ± 3.21	5.47 ± 2.93	0.12
CIMT	0.61 ± 0.1	0.70 ± 0.1	0.00	0.71 ± 0.1	0.78 ± 0.2	0.00
FMD	17.08 ± 7.9	15.35 ± 8.2	0.28	14.28 ± 7.56	12.72 ± 6.84	0.28
ABI	1.17 ± 0.1	1.15 ± 0.1	0.36	1.14 ± 0.10	1.12 ± 0.11	0.32

The serum levels of inflammatory mediators of IL-6 and, hs-CRP were evaluated in these four groups and we found that there was an increasing trend in serum concentrations of these mediators, in a way that control-non-hypertensive patients had the lowest and diabetic- hypertensive subjects presented with the highest levels of IL-6 and hs-CRP and the two other groups were placed between. However this difference did not reach a significant level.

**Figure 1:**
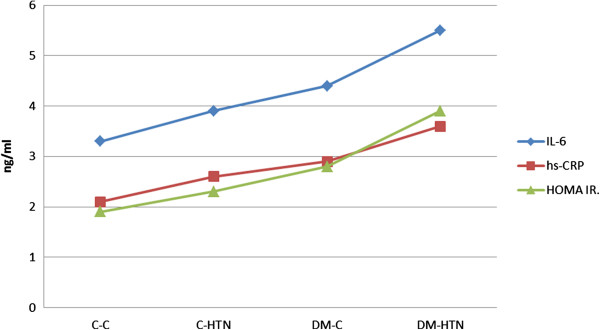
**Insulin resistance status and levels of inflammatory mediators in diabetic and control groups.** C-C = control normotensive; C-HTN = control hypertensive; DM-C = diabetic normotensive; DM-HTN = diabetic hypertensive.

When we looked at the insulin resistance state of these four subgroups, we observed a similar pattern on which control-non-hypertensive subjects having the lowest (HOMA IR = 1.93 ± 1.39) and diabetic hypertensive having the highest (HOMA IR = 3.89 ± 2.99) HOMA IR (Figure 1).

Looking at the subclinical atherosclerosis measures revealed that in both control and diabetic groups, only CIMT measure was significantly different between subgroups of non-hypertensive and hypertensive subjects. The trend of decrease in FMD and ABI values was from control-non- hypertensive to control-hypertensive to diabetic non-hypertensive and finally to diabetic hypertensive subjects.

Evaluation of the association between atherosclerosis markers CIMT, FMD and ABI with various parameters, using linear regression analysis revealed different results in diabetic and non-diabetic subjects. In non-diabetic, a constant significant association existed between age and CIMT, ABI and FMD, while in diabetic, age only correlated with CIMT and not with the other two markers. Table 3 presents the relation between these surrogate markers with various parameters.

**Table 3:** Linear regression analysis for evaluation of association of CIMT, FMD and ABI with different parameters in diabetic and non-diabetic subjects

**Variable**	**CIMT**		**FMD**		**ABI**	
	**Non diabetic β, (P)§**	**Diabetic β, (P)§**	**Non diabetic β, (P)§**	**Diabetic β, (P)§**	**Non diabetic β, (P)§**	**Diabetic β, (P)§**
Age	0.33 (0.01)	0.31 (0.01)	-0.23 (0.02)	-0.15 (0.12)	0.19 (0.04)	-0.09 (0.36)
Sex	-0.12 (0.2)	-0.23 (0.01)	0.09 (0.38)	0.19 (0.06)	0.03 (0.73)	-0.16 (0.1)
Systolic BP	0.27 (0.01)	0.14 (0.12)	0.32 (0.01)	-0.04 (0.72)	-0.01 (0.31)	0.03 (0.8)
Diastolic BP	0.22 (<0.01)	0.19 (0.03)	-0.13 (0.19)	-0.05 (0.61)	-0.16 (0.08)	0.06 (0.54)
BMI	-0.07 (0.45)	-0.21 (0.04)	-0.13 (0.2)	-0.05 (0.65)	0.20 (0.04)	0.01 (0.94)
Waist circumference	-0.001 (0.98)	-0.24 (0.01)	0.05 (0.64)	0.04 (0.70)	0.11 (0.28)	0.00 (0.99)
HOMA IR	-0.02 (0.78)	-0.12 (0.2)	-0.17 (0.09)	0.00 (1.00)	0.02 (0.81)	-0.03 (0.75)
Hs-CRP	-0.01 (0.95)	0.00 (0.97)	-0.16 (0.1)	0.10 (0.37)	-0.15 (0.09)	-0.09 (0.33)
IL-6	0.18 (0.03)	-0.07 (0.43)	-0.20 (0.81)	0.13 (0.21)	-0.15 (0.11)	0.08 (0.43)

BMI = body mass index; BP = blood pressure; HOMA IR = Homeostatic model assessment; hs- CRP = high sensitive C-reactive protein; IL-6 = interleukin 6; CIMT = Carotid Intima Media thickness; FMD = flow Mediated Dilatation; ABI = Ankle - brachial index. §: adjusted for age, sex, triglyceride, total cholesterol, HDL-C and LDL-C.

For assessment of effect of hypertension on subclinical atherosclerosis we categorized CIMT, FMD and ABI values into 4 groups based on 75% better values and 25% worse values of diabetic and control groups and odds ratios (OR) of worse value were calculated in multiple logistic regression models. In this model hypertension was recognized as a risk factor for increasing CIMT (OR = 2.93 (1.03-8.33), however similar scenario was not observed for diminution of FMD and ABI (Table 4).

**Table 4:** Hypertension as risk factor of subclinical atherosclerosis

	**Diabetic**		**Control**	
	**Adjusted OR (CI)**	**P**	**Adjusted OR (CI)**	**P**
CIMT (75%)	3.15 (1.03-9.56)	0.04	2.93 (1.03-8.33)	0.04
FMD (75%)	0.80 (0.22-2.88)	0.73	2.44 (0.72-8.25)	0.15
ABI (75%)	1.07 (0.39-2.91)	0.39	1.58 (0.54-4.57)	0.89

OR: Odds Ratio, CI: Confidence Interval, CIMT: Carotid Intima-Media Thickness, FMD: Flow Mediated Dilation, ABI: Ankle Brachial Index.

Adjusted for Age, sex, hypertension duration, diabetes mellitus duration, cholesterol, triglyceride and HDL-C.

## Discussion

CIMT, FMD and Peripheral artery disease (PAD), evaluated by ABI, have been related by several studies to cardiovascular risk factors and events and their non-invasive measurement makes it possible to more easily detect the initial phases of atherosclerosis [16].

In this study first we showed that compared with non-diabetic subjects, atherosclerosis was significantly advanced in non-complicated diabetic subjects as this process was assessed by three surrogate markers of CIMT, FMD, and ABI. To our knowledge few studies has done so by simultaneous characterization of structural, functional and peripheral vascular alterations in T2DM subjects without apparent macrovascular disease. Our results are in line with previous studies in which increased CIMT [17], an impaired FMD and ABI [18,19] were indicated in diabetic subjects compared to control individuals. Interestingly, in a systematic review involving.

4019 diabetic subjects, an estimated mean difference of 0.13 mm in IMT was observed between diabetic and control group, a value that is similar to what we have seen in our subjects [16]. In diabetes the accelerated vascular disease is not restricted to the coronary circulation but also occurs in peripheral vascular systems. In general population an ABI less than 0.9 is regarded as an indicator of PAD and has a high sensitivity and specificity to identify an obstruction greater than 50% in the vasculature of the lower limbs [20]. The cut-off values for the highest sensitivity and specificity for PAD in diabetic patients, however, might be different. In our study the mean of ABI in both groups was over 0.9 though diabetic subjects still had significantly lower ABI than control. This could be translated into that the need for intensive treatment of risk factors and initiation of antiplatelet therapy in diabetic even without apparent PAD should be considered.

In this study we showed that our hypertensive subjects regardless of being diabetic or non- diabetic had higher CIMT. Similarly, Mahfouz et al. [21] and Sorof et al. [22] showed the greater value of CIMT in hypertensive subjects. Moreover, Post-hoc analyses of the association between antihypertensive treatment and CIMT in the Troglitazone Atherosclerosis Regression Trial (TART), which assessed CIMT progression in adults with insulin-treated type 2 diabetes, found that higher systolic blood pressure was associated with a higher CIMT progression rate [23]. Elevation of blood pressure has been shown to be associated with several alterations in the vascular system, including enhancement of vascular tone, increased shear stress, and activation of the sympathetic nervous and renin-angiotensin aldosterone systems. Because these functional and structural changes promote endothelial damage and lead to atherosclerosis, hypertension is one of the main causes of endothelial dysfunction [24]. In this study we did demonstrate favorable trends for FMD and ABI impairment in hypertensive versus non-hypertensive subjects, although these changes did not reach a significant level. Even though we cannot clearly explain this observation, but one explanation for lack of seeing a remarkable difference would be that since the effect of hypertension on arteries is not similar on all vessels due to geometrical and oscillating shear stress properties of sites, it is predictable that hypertension produce more effect on carotid rather than limb vessels which are represented by impaired FMD and ABI [25]. Lack of difference in ABI might be also related to the fact that ABI is a late-onset finding in hypertensive subjects and as was demonstrated in ABI values, none of our subjects had ABI less than 0.9, a value that is the indicator of PAD.

A close relation between hypertension and inflammation is reported by investigators [26,27]. Pathophysiologically, inflammation has been implicated in both endothelial dysfunction and arterial stiffness in hypertension and it is assumed that every mechanism leading to inflammation will accelerate this process [27].

Here we detected an increasing trend in two basic inflammatory mediators of IL-6 and CRP in hypertensive subjects no matter they were in control or diabetic group. Even though our study was not powered enough to detect the significant change, our data is compatible with others and emphasizes on the link between hypertension and inflammation, but is insufficient to reveal whether the primary inflammation is the underlying cause of hypertension or inflammation is a product of being hypertensive.

One main finding of this study was that we demonstrated that hypertension is an independent risk factor of atherosclerosis in diabetes, which produces a significant additive effect with diabetes mellitus on elevation of IMT but not FMD or ABI. this does not mean that hypertension did not affect the endothelial function or arterial resistance in diabetic subjects, but we could assume that the presence of diabetes per se is of so crucial importance for the development of atherosclerosis (due to development and persistence of a cluster of multiple interrelated metabolic disturbances), that it over shadows the contribution of other risk factors.

A number of risk factors have been associated with the development of atherosclerosis in the carotid arteries. Generally advanced age, hypertension, cigarette smoking, hyperlipidemia and diabetes are the known factors [28]. In our study, the only persistent factor that affects all the surrogate markers of atherosclerosis in normal subjects but not the diabetic was age. On the other hand, in diabetic subjects, in addition to age and blood pressure, BMI also had an effect on CIMT. Such a difference between factors related to atherosclerosis in diabetic and control subjects were also observed for FMD and ABI. Studies of the correlation between risk factors of atherosclerosis and surrogate markers of this process in different populations have produced different results, with some showing an association between risk factors and atherosclerosis markers but others finding no relationship at all. Similar to our study, Butt and Zakaria found an association between BMI and CIMT in diabetic [17], while Taniguchi et al. [29] found no correlation with BMI. Association of BMI with increased CIMT may suggest that central fat accumulation could accelerate the development of earlier clinically silent stages of atherosclerosis.

In conclusion we found that hypertension could produce an additive effect on increased CIMT but not on FMD and ABI on diabetic subjects. Indeed, we showed that markers of atherosclerosis were associated with different risk factors in diabetic and control subjects. We believe that our finding support the possibility that in diabetic subjects CIMT, FMD and ABI provide independent information about the atherosclerotic process. Atherosclerosis is a complex disease and may have complex pathways in different population. Further studies in larger scale are required to better explain the interrelation and contribution of various risk factors of atherosclerosis in diabetic in comparison to control subjects.

## Abbreviations

T2DM: Type 2 diabetes mellitus; CAD: Coronary artery disease; CIMT: Carotid intima-media thickness measurement; FMD: Brachial artery flow-mediated dilatation test; ABI: Ankle-brachial index; EMRI: Endocrinology and metabolism research institute; FBS: Fasting plasma sugar; TG: Triglyceride; HDL: High density lipoprotein cholesterol; LDL: Low density lipoprotein cholesterol; BUN: Blood urea nitrogen; hs-CRP: High sensitive C-reactive protein; HOMA: Homeostasis model assessment; ANOVA: Univariate analysis of variance; PAD: Peripheral artery disease; TART: Troglitazone atherosclerosis regression trial.

## Competing interests

The authors declare that they have no competing interests.

## Authors’ contributions

SA: drafted and edited the main manuscript, HF: the main investigator of the study, conducted the study, and approved the final version of the manuscript, FS: performed the interpretation and analysis of data, AM: performed the cardiovascular assessment, MM: assisted in data collection, ZB: performed the patient recruitment and data collection, YT: assisted in data collection and cardiovascular assessment. All authors read and approved the final manuscript.
